# National evidence linking robotic total knee arthroplasty to reduced 90-day readmissions, complications, and readmission costs

**DOI:** 10.1186/s42836-026-00373-y

**Published:** 2026-02-28

**Authors:** David Maman, Yaniv Steinfeld, Yaron Berkovich

**Affiliations:** 1 Carmel Medical Center, 3436212 Haifa, Israel; 2 Faculty of Medicine, Technion Israel Institute of Technology, 2611001 Haifa, Israel

**Keywords:** Total knee arthroplasty, Post-operative complications, Robotic knee surgery, NRD

## Abstract

**Purpose:**

To compare 90-day readmissions, complications, and resource use after robotic-assisted versus conventional total knee arthroplasty (TKA) in a contemporary, nationally representative cohort.

**Methods:**

Retrospective cohort study using the Nationwide Readmissions Database (NRD) 2020–2022. Primary TKA identified from the PR1 field (ICD-10-PCS). Exclusions included non-elective admissions, revisions, bilateral procedures, age < 18, oncology/fracture/reoperation, COVID-19, and discharges after September. Readmissions within 90 days were categorized (prosthesis/SSI, mechanical/implant, VTE). 1:1 propensity score matching (nearest neighbor, caliper 0.01, no replacement) included demographics, comorbidities, hospital factors, and year.

**Results:**

After matching, 96,982 patients (48,491 per group) were analyzed. Robotic TKA showed lower all-cause 90-day readmission (5.0% vs 6.5%), superior readmission-free survival (log-rank *P* < 0.001), shorter readmission LOS (4.8 vs 5.6 days), and lower readmission charges ($66,769 vs $75,544), with slightly higher index charges ($78,125 vs $74,090). Risks were lower for VTE/PE, pneumonia, transfusion, postoperative pain, and prosthesis/SSI-related and mechanical readmissions.

**Conclusions:**

In the largest contemporary national analysis, robotic TKA was associated with fewer early complications, lower 90-day readmissions, and reduced readmission resource use compared with conventional TKA.

**Supplementary Information:**

The online version contains supplementary material available at 10.1186/s42836-026-00373-y.

## Introduction

Total knee arthroplasty (TKA) is one of the most frequently performed orthopedic procedures worldwide [1], with demand in the United States projected to exceed three million annual cases by 2030 [2, 3]. Despite advances in implant design, perioperative protocols, and rehabilitation pathways, early complications and unplanned readmissions remain a persistent burden for patients and healthcare systems operating under value-based reimbursement models [4–7]. Even small reductions in 90-day readmission rates can translate into substantial clinical and financial impact at the population level [8].

Robotic-assisted TKA (RA-TKA) has emerged as a technology designed to improve surgical accuracy, optimize implant positioning, and achieve more consistent soft-tissue balance [9–11]. Institutional series and registry reports have suggested lower complication rates [12] and improved functional recovery with robotic systems [13], but national-level evidence remains limited. In particular, the effect of RA-TKA on 90-day readmission, a key quality metric in bundled payment models, has not been comprehensively assessed using large, contemporary data [14]. Prior analyses were often restricted to in-hospital outcomes, earlier datasets, or relatively small cohorts, leaving uncertainty as to whether perioperative benefits translate into meaningful reductions in early readmissions [15].

The present study leverages the most up-to-date Nationwide Readmissions Database (NRD, 2020–2022) to address this gap. By restricting index admissions to January–September discharges to ensure complete 90-day follow-up, applying rigorous propensity score matching across demographic, clinical, and hospital characteristics, and grouping readmission diagnoses into clinically meaningful categories, this analysis provides the largest and most comprehensive national assessment of RA-TKA and 90-day readmission to date.

The null hypothesis was that there would be no difference in 90-day readmission rates, postoperative complications, or readmission-related resource utilization between robotic-assisted and conventional TKA.

## Materials and methods

### Database and study period

We conducted a retrospective cohort study using the Nationwide Readmissions Database (NRD), 2020–2022, developed by HCUP. The NRD is a nationally representative, all-payer database specifically designed to evaluate readmissions. Each year is released as a separate dataset; therefore, only procedures performed from January through September were included to allow for a complete 90-day follow-up. The 2022 dataset, the most recent available release, was incorporated, making this the most contemporary nationwide analysis to date.

### Cohort identification and exclusions

**Fig. 1:**
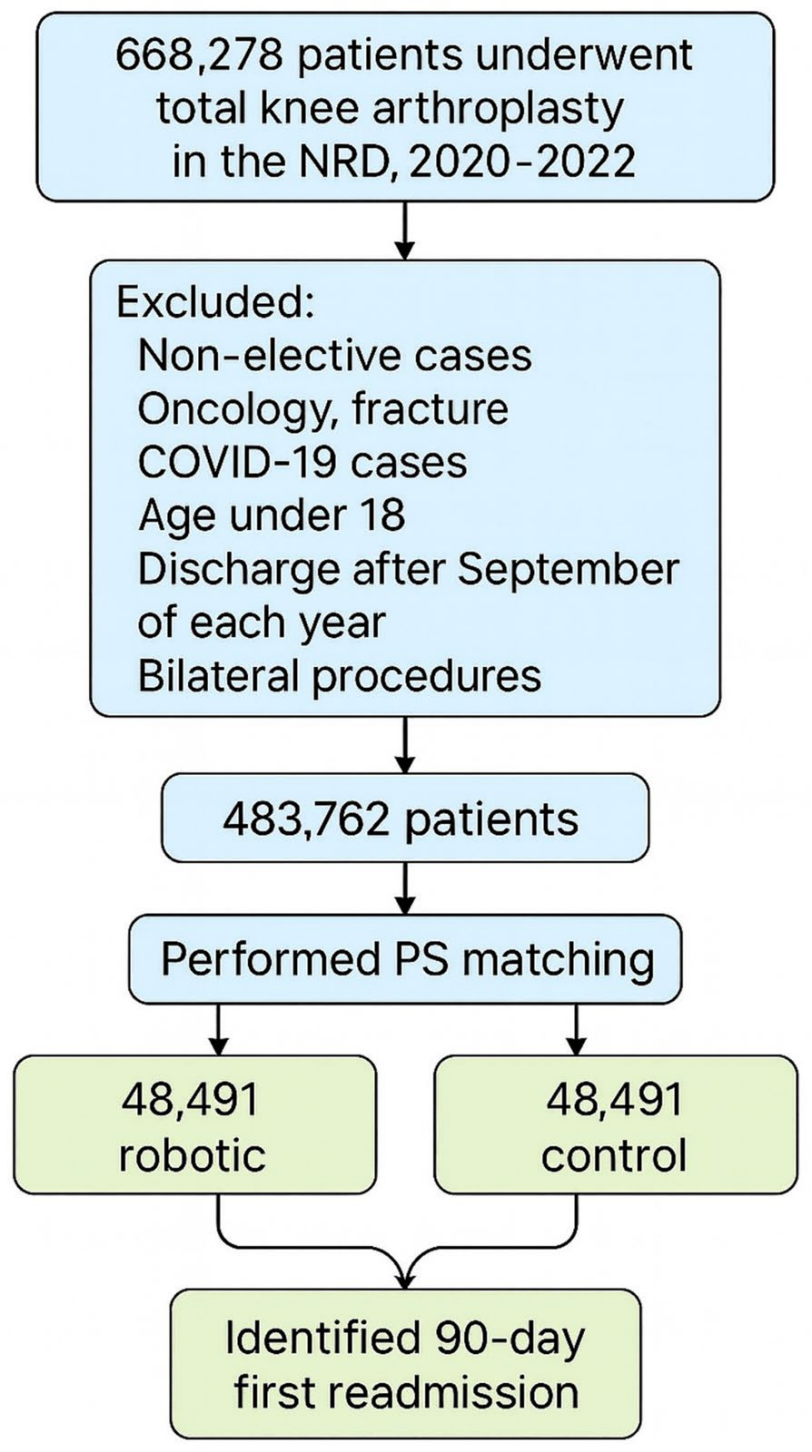
Flowchart of patient selection, exclusions, and propensity score matching for robotic versus conventional total knee arthroplasty with identification of 90-day readmissions

The patient selection process is outlined in Fig. 1. Primary total knee arthroplasty cases were identified from the primary procedure field (I10_PR1) using standardized ICD-10-PCS replacement codes for primary knee arthroplasty, as detailed in Supplementary Table S1. Robotic-assisted procedures were identified using ICD-10-PCS robotic assistance modifier codes, also listed in Supplementary Table S1.

Non-elective admissions were excluded, as were cases involving revision total knee arthroplasty at the index admission, bilateral procedures, and patients younger than 18 years. Admissions related to oncology, fracture, or reoperation were excluded to avoid confounding clinical indications. Any case coded for COVID-19 infection was also excluded. In addition, index hospitalizations with discharge occurring after September of each year were excluded to ensure a complete 90-day follow-up. Patients readmitted for elective contralateral TKA were excluded to avoid misclassification as complications. Only cases where the index TKA clearly preceded any readmission were retained.

### Study outcomes

The primary endpoint of the study was hospital readmission within 90 days following the index total knee arthroplasty. Prosthesis-related or surgical site infection readmissions included prosthesis infections or inflammatory reactions, surgical site infections, and lower limb cellulitis. Mechanical or implant-related readmissions included non-infectious complications related to the prosthesis or implant loosening. Venous thromboembolism-related readmissions included deep vein thrombosis and pulmonary embolism. All ICD-10-PCS procedure codes recorded during readmission were screened to identify surgical interventions, and elective contralateral total knee arthroplasty readmissions were excluded to avoid misclassification as postoperative complications.

Secondary endpoints included perioperative outcomes, specifically hospital length of stay and total hospital charges during the index admission. Postoperative complications were evaluated and reported separately as those without statistically significant differences between groups and those demonstrating significant differences.Additional readmission-related outcomes included length of stay during readmission, hospital charges associated with readmission, time to readmission, in-hospital mortality during readmission, and all-cause 90-day readmission.

### Propensity score matching

Logistic regression models were used to generate propensity scores incorporating patient demographics, including age and sex; comorbid conditions such as hypertension, dyslipidemia, obstructive sleep apnea, chronic kidney disease, type 2 diabetes, congestive heart failure, chronic anemia, chronic lung disease, obesity, osteoporosis, Parkinson’s disease, history of myocardial infarction, history of cerebrovascular accident, thyroid disorders, and liver disease; hospital characteristics including bed size, teaching status, and location; and year of surgery (2020–2022).

Matching was performed with a nearest-neighbor algorithm, caliper 0.01, no replacement. Covariate balance was confirmed using standardized mean differences (SMD < 0.1).

### Analytical approach

Continuous variables are reported as means with standard deviations and were compared between groups using independent *t*-tests. Categorical variables are presented as counts and percentages and were compared using chi-square tests. Relative risks (RRs) with 95% confidence intervals were calculated for postoperative complications and cause-specific 90-day readmissions; RRs were defined as the risk in the conventional (non-robotic) TKA group divided by the risk in the robotic TKA group, such that an RR greater than 1 indicates a higher risk associated with conventional surgery. Time to readmission was evaluated using Kaplan–Meier survival analysis, with group differences assessed by the log-rank test; patients were censored at death, beyond 90 days from the index discharge, or at the end of the database year. All statistical analyses were performed using SPSS (IBM, Armonk, NY) and MATLAB (MathWorks, Natick, MA), and figures were generated using both platforms. A two-sided *P*-value < 0.05 was considered statistically significant. All analyses incorporated NRD sampling weights to generate nationally representative estimates.

### Ethical aspects

The NRD contains only de-identified discharge data. This study was therefore exempt from institutional review board approval and informed consent.

## Results

### Baseline characteristics

A total of 96,982 patients were included following propensity score matching, with 48,491 undergoing robotic-assisted TKA and 48,491 undergoing conventional TKA. Baseline demographic and clinical characteristics were well balanced between groups, as demonstrated by standardized mean differences (SMD) below 0.10 for all variables. Age, sex distribution, year of surgery, and major comorbid conditions, including hypertension, dyslipidemia, diabetes, chronic kidney disease, and obesity, were comparable between groups, confirming successful propensity score matching (Table 1).

**Table 1:** Baseline characteristics after propensity score matching

Parameter	Non-robotic surgery	Robotic surgery	SMD
Total surgeries	48,491 (50%)	48,491 (50%)	-
Average age (y)	67.8	67.8	0.01
Female (%)	60.6	61.0	0.01
Year—2020 (%)	34.3	34.7	0.01
Year—2021 (%)	34.9	34.5	0.01
Year—2022 (%)	30.8	30.7	0.00
Hypertension (%)	56.9	57.1	0.00
Dyslipidemia (%)	52.9	52.8	0.00
Obstructive sleep apnea (%)	15.8	15.3	0.01
Chronic anemia (%)	4.8	4.8	0.00
Osteoporosis (%)	4.7	4.9	0.01
Parkinson disease (%)	0.8	0.8	0.00
Chronic kidney disease (%)	9.2	9.2	0.00
Type 2 diabetes (%)	22.2	21.5	0.02
Congestive heart failure (%)	1.3	1.4	0.01
Chronic lung disease (%)	5.7	5.4	0.01
Disorders of thyroid (%)	17.7	18.1	0.01
Liver disease (%)	1.9	1.8	0.01
History of myocardial infarction (%)	3.4	3.2	0.01
History of cerebrovascular Accident (%)	4.2	4.2	0.00
Obesity (%)	36	35.5	0.01

Standardized mean differences (SMD) are provided to demonstrate post-match covariate balance (SMD < 0.10 considered acceptable)

### Perioperative outcomes

Perioperative measures are summarized in Table 2. Patients in the robotic-assisted TKA group had a significantly shorter mean length of stay compared with the conventional TKA group (2.0 vs 2.2 days, *P* < 0.01). However, robotic TKA was associated with slightly higher mean hospital charges ($78,125 vs $74,090, *P* < 0.01).

**Table 2:** Perioperative outcomes of patients undergoing robotic versus conventional total knee arthroplasty

	**Non-robotic surgery**	**Robotic surgery**	**Significance**
Length of stay means in days	2.2 (Std. deviation 2.4)	2.0 (Std. deviation 2.5)	*P* < 0.01
Total charges mean in $	74,090 (Std. Deviation 50,009)	78,125 (Std. deviation 52,234)	*P* < 0.01

### Postoperative complications without statistically significant differences between robotic and conventional total knee arthroplasty

Table 3 presents postoperative complications for which no statistically significant differences were observed between groups. Intraoperative fracture occurred in 0.36% of conventional TKA versus 0.32% of robotic TKA (*P* = 0.26). Urinary tract infection was observed in 0.72% versus 0.64% (*P* = 0.14), and sepsis in 0.07% versus 0.05% (*P* = 0.25).

**Table 3:** Postoperative complications without statistically significant differences between robotic and conventional total knee arthroplasty

Parameter	Non-robotic surgery	Robotic surgery	Significance
Intraoperative Fracture	0.36%	0.32%	*P* = 0.26
UTI	0.72%	0.64%	*P* = 0.14
Sepsis	0.07%	0.05%	*P* = 0.25

### Postoperative complications with significant differences

**Fig. 2:**
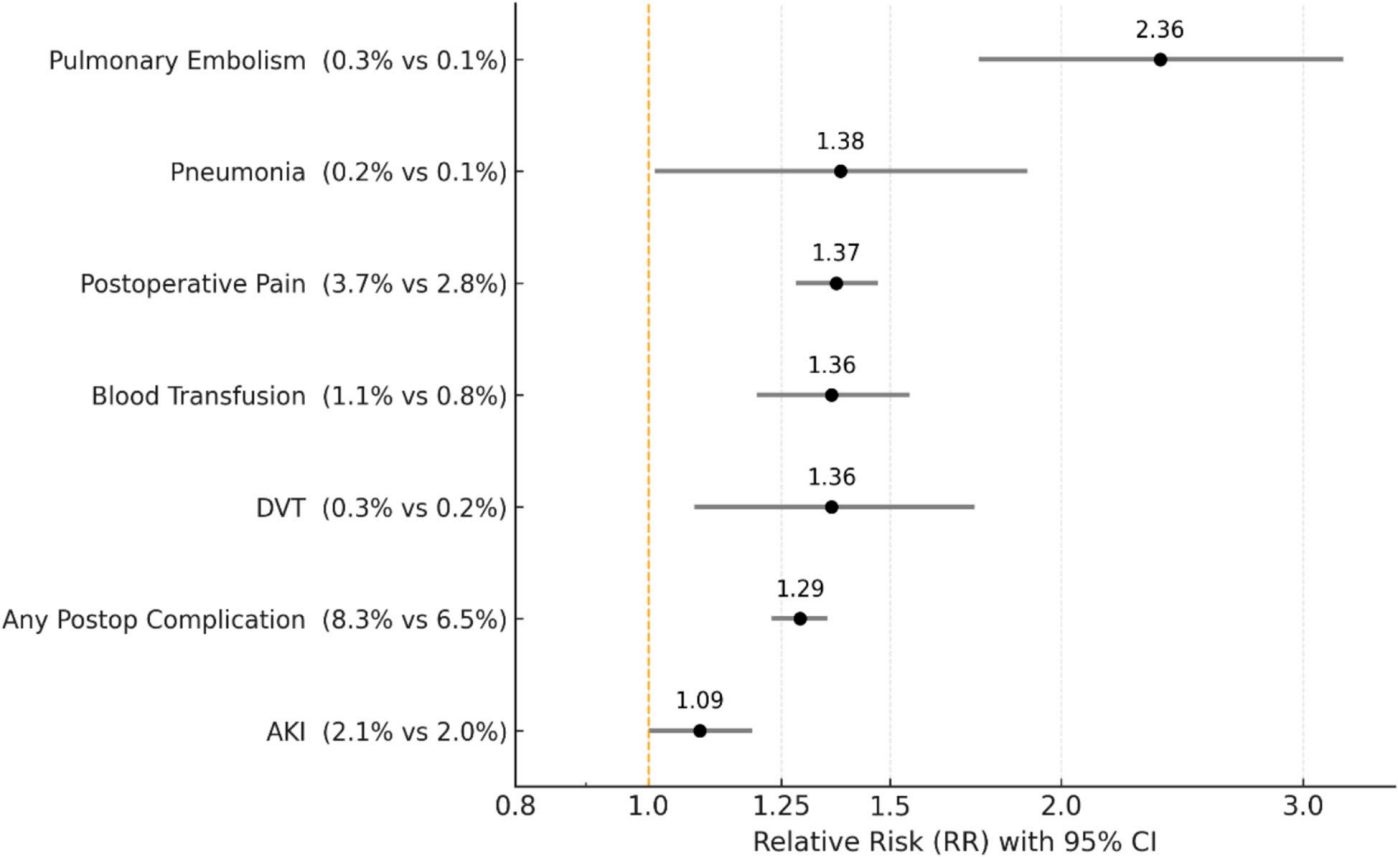
Forest plot showing elevated risk of postoperative complications in non-robotic compared to robotic TKA

Figure 2 summarizes postoperative complications that differed significantly between groups. Overall, conventional TKA was associated with higher complication rates compared with robotic-assisted TKA. Acute kidney injury occurred more frequently after conventional TKA (2.1%) than after robotic TKA (2.0%; RR 1.09, 95% CI 1.00–1.19, *P* = 0.04). The composite outcome of any postoperative complication was also higher in the conventional group (8.3% vs 6.5%; RR 1.29, 95% CI 1.23–1.35, *P* < 0.01). Conventional TKA was associated with increased risks of deep vein thrombosis (0.3% vs 0.2%; RR 1.36, 95% CI 1.08–1.73, *P* < 0.01), blood transfusion (1.1% vs 0.8%; RR 1.36, 95% CI 1.20–1.55, *P* < 0.01), and pneumonia (0.2% vs 0.1%; RR 1.38, 95% CI 1.01–1.89, *P* = 0.04). Postoperative pain was more commonly documented following conventional TKA (3.7% vs 2.8%; RR 1.37, 95% CI 1.28–1.47, *P* < 0.01). In addition, pulmonary embolism occurred significantly more often after conventional TKA compared with robotic TKA (0.3% vs 0.1%; RR 2.36, 95% CI 1.74–3.21, *P* < 0.01).

### Ninety-day readmission outcomes

Ninety-day readmission outcomes are summarized in Table 4. Patients in the robotic TKA group had a significantly shorter mean length of stay during readmission (4.8 vs 5.6 days, *P* < 0.01) and lower mean hospital charges ($66,769 vs $75,544, *P* < 0.01). The time to readmission did not differ significantly between groups (30.5 vs 31.2 days, *P* = 0.32). Mortality during readmission was lower in robotic TKA patients (0.06% vs 0.08%, *P* < 0.01). Additionally, all-cause 90-day readmission was significantly reduced in the robotic group (5.0% vs 6.5%, *P* < 0.01).

**Table 4:** Ninety-day readmission outcomes following robotic versus conventional total knee arthroplasty

	**Non-robotic surgery**	**Robotic surgery**	**Significance**
Length of stay of Readmission mean in days	5.6 (Std. deviation 7.1)	4.8 (Std. deviation 4.7)	*P* < 0.01
Total charges of Readmission mean in $	75,544 (Std. Deviation 154,739)	66,769 (Std. deviation 80,730)	*P* < 0.01
Time to Readmission in days	31.2 (Std. Deviation 26.9)	30.5 (Std. deviation 25.6)	*P* = 0.32
Mortality in Readmission	0.08%	0.06%	*P* < 0.01
All-Cause Readmission	6.5%	5.0%	*P* < 0.01

### Kaplan–Meier survival analysis

**Fig. 3:**
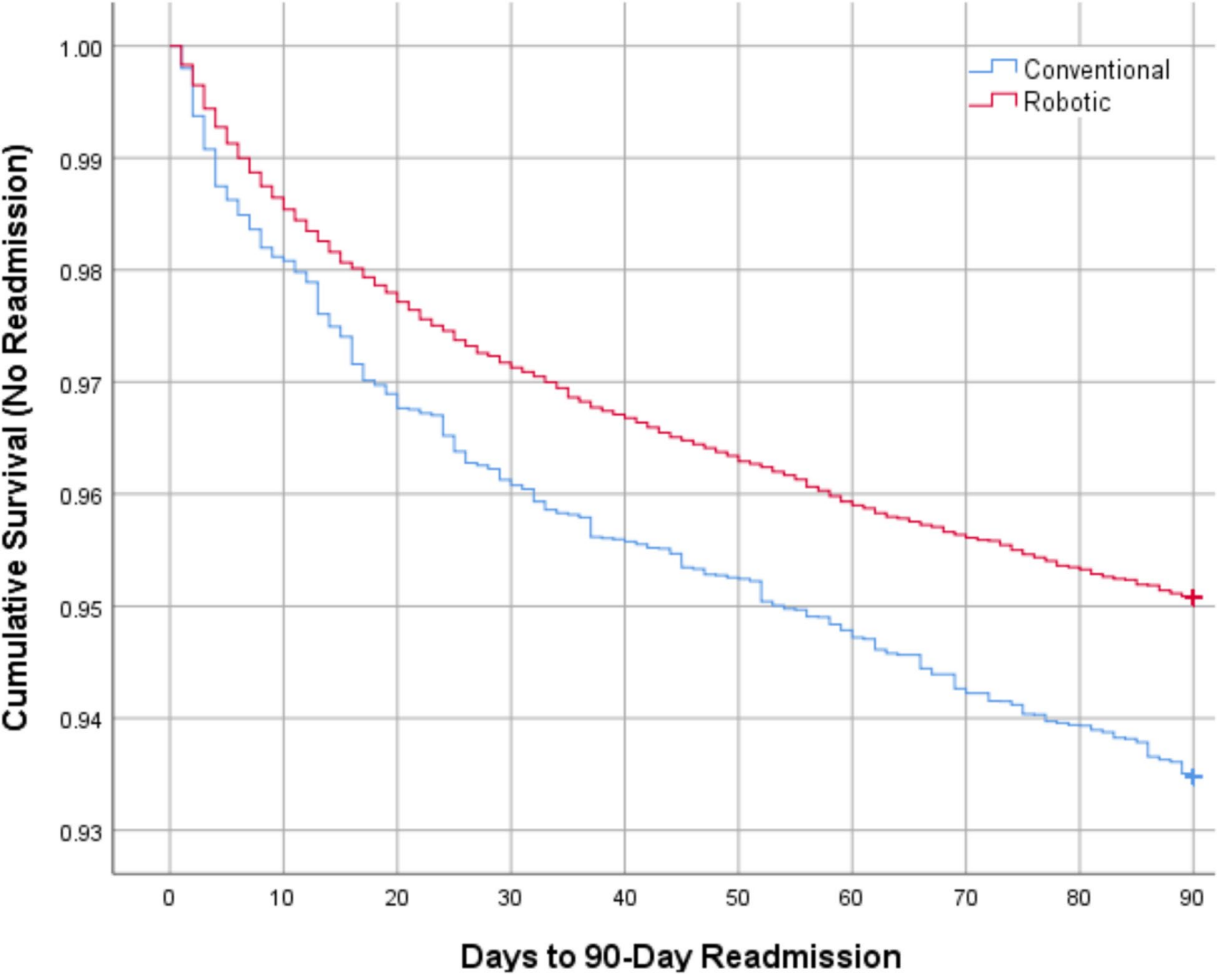
Kaplan–Meier survival curves showing 90-day readmission-free survival following robotic versus conventional total knee arthroplasty. Robotic TKA demonstrated significantly better readmission-free survival (*P* < 0.001, log-rank test)

Kaplan–Meier survival analysis demonstrated a significantly higher readmission-free survival in the robotic TKA group compared with the conventional group (log-rank *P* < 0.001). By 90 days, cumulative survival without readmission remained consistently higher among patients who underwent robotic TKA (Fig. 3).

### Ninety-day readmission causes

**Fig. 4:**
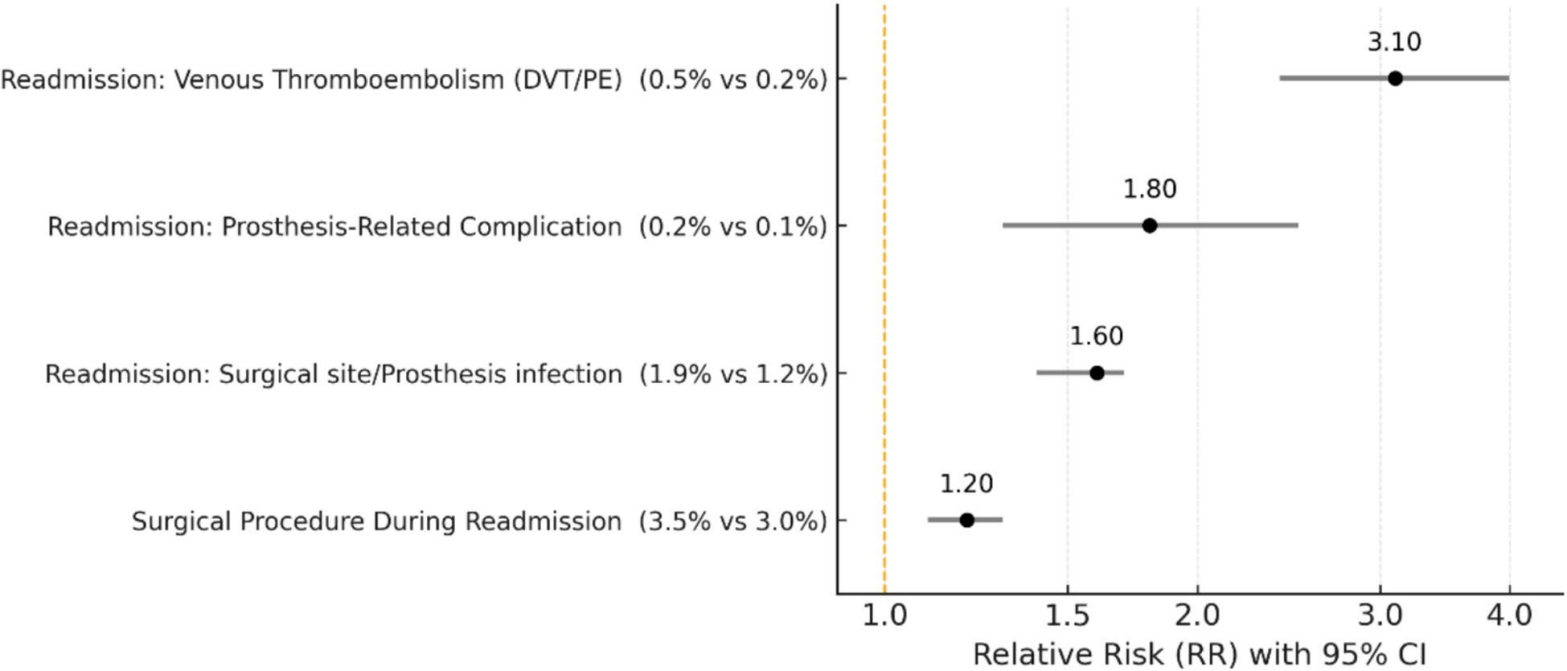
Forest plot of 90-day readmission causes comparing robotic versus conventional total knee arthroplasty

Figure 4 presents relative risks for specific causes of readmission within 90 days (RR defined as conventional/robotic). Surgical procedures during readmission occurred more often after conventional TKA (3.5%) than robotic TKA (3.0%; RR 1.2, 95% CI 1.1–1.3). Readmissions for surgical site infection or prosthesis-related infection were higher after conventional TKA (1.9%) than robotic TKA (1.2%; RR 1.6, 95% CI 1.4–1.7). Prosthesis-related complications occurred in 0.2% after conventional TKA versus 0.1% after robotic TKA (RR 1.8, 95% CI 1.3–2.5). Venous thromboembolism (DVT/PE) readmissions were observed in 0.5% after conventional TKA versus 0.2% after robotic TKA (RR 3.1, 95% CI 2.4–4.0).

## Discussion

### Principal findings

In this national, propensity-matched cohort of nearly 97,000 patients, robotic-assisted total knee arthroplasty was consistently associated with superior early outcomes across multiple clinically relevant dimensions. Compared with conventional TKA, robotic assistance was associated with a significantly lower all-cause 90-day readmission rate (5.0% vs 6.5%) and improved readmission-free survival on Kaplan–Meier analysis (log-rank *P* < 0.001). Among patients who required readmission, robotic-assisted TKA was associated with a shorter length of stay (4.8 vs 5.6 days) and substantially lower readmission-related hospital charges, despite slightly higher charges during the index hospitalization. In addition, robotic TKA was associated with reduced rates of several important postoperative complications, including venous thromboembolism, pulmonary embolism, pneumonia, blood transfusion, and postoperative pain. Cause-specific analyses further demonstrated lower risks of major readmission diagnoses following robotic TKA, particularly prosthesis-related infection, mechanical complications, and thromboembolic events.

### Interpretation and clinical significance

The reduction in thromboembolic and pulmonary complications may reflect less intramedullary canal violation, more controlled bone resection, and potentially earlier mobilization associated with robotic workflows [16]. Lower rates of infection-related readmissions align with the hypothesis that robotic precision reduces soft-tissue trauma and improves wound handling [17, 18]. Importantly, the observed absolute risk reduction of 1.5% in 90-day readmission equates to a number needed to treat of ~ 67, a clinically meaningful effect when applied to the scale of national arthroplasty volumes.

From a health systems perspective, the observed reduction in readmission charges of nearly $9000 per case offsets much of the modest increase in index hospitalization charges [19–21]. This suggests that the adoption of RA-TKA could be cost-neutral or even cost-saving within bundled payment models, particularly in high-volume centers where robotic workflows are most efficient [4, 22, 23].

### Comparison with existing literature

Most prior national studies of RA-TKA were limited to in-hospital outcomes or earlier datasets [23–25]. By contrast, our analysis uses the latest NRD release (2022)**,** includes robust exclusions such as contralateral elective TKA, and provides the first large-scale breakdown of cause-specific 90-day readmissions [26]. Our findings extend earlier reports by confirming not only perioperative benefits but also significant differences in post-discharge outcomes, critical metrics for both patients and payers [27].

Institutional studies and registries have suggested improvements in alignment, functional scores, and satisfaction with RA-TKA [24]. This analysis complements those findings by showing that such technical precision is associated with measurable reductions in early complications and readmissions at the population level [28, 29].

### Strengths and limitations

The strengths of this study include its scale, recency, and methodological rigor: strict identification of primary TKA procedures, exclusion of confounding cases, and propensity score matching across demographic, clinical, and hospital-level variables. The use of Kaplan–Meier survival analysis and cause-specific forest plots provides a granular view of outcomes that enhances interpretability [30, 31].

Limitations include the inherent constraints of administrative coding and the potential for residual confounding. Robotic assistance was identified using ICD-10-PCS codes (8E0Y0CZ/8E0YXCZ); these exposure codes have limited external validation in NRD-based arthroplasty research, and coding practices may vary across hospitals, creating the possibility of misclassification that could bias estimates in either direction. Although we performed 1:1 propensity score matching across demographics, comorbidities, hospital characteristics, and year, important clinical and technical variables are not available in NRD (e.g., continuous BMI/obesity severity, ASA class, functional status, deformity severity, implant type/constraint, operative time, and surgeon/hospital volume), and these unmeasured factors may influence both procedure selection and outcomes. In addition, NRD captures inpatient hospitalizations and does not include same-day/outpatient arthroplasty performed in ambulatory settings; given the ongoing shift of lower-risk TKA to outpatient pathways, our cohort may represent a relatively higher-acuity inpatient subset, which may limit generalizability and could bias effect estimates depending on patterns of robotic adoption. Finally, NRD reports hospital charges rather than true costs or payments [32]; because cost-to-charge ratios were not applied, these figures should be interpreted as relative comparisons of billed resource utilization rather than absolute economic valuations.

### Implications and future directions

These findings suggest that RA-TKA not only improves perioperative safety but also reduces clinically and financially meaningful 90-day readmissions. For policymakers and payers, RA-TKA may represent a technology that aligns with value-based care principles by lowering complication-related expenditures. For clinicians, the data support the broader adoption of RA-TKA as part of strategies to improve early recovery and reduce the burden of unplanned readmission.

Future research should focus on integrating administrative data with arthroplasty registries to assess long-term revision rates, patient-reported outcomes, and cost-effectiveness. Subgroup analyses may identify patients, such as those with severe deformities, obesity, or complex comorbidities, who derive the greatest incremental benefit from robotic assistance.

## Conclusion

In the largest and most contemporary national analysis to date, robotic-assisted TKA demonstrated lower 90-day readmission rates, fewer early complications, and reduced readmission resource use compared with conventional TKA. These findings suggest that robotic precision is translating into real-world clinical and economic benefits, reinforcing RA-TKA’s role in advancing the quality and value of knee arthroplasty.

## Supplementary Information

Supplementary Material 1.

## Data Availability

This study used publicly available data from the HCUP Nationwide Readmissions Database (NRD). Data can be obtained directly from HCUP (https://hcup-us.ahrq.gov/dataoverview.jsp) upon purchase.
