# Supplementary material for: National evidence linking robotic total knee arthroplasty to reduced 90-day readmissions, complications, and readmission costs
Source: Arthroplasty. 2026 Feb 28;8:16. doi: 10.1186/s42836-026-00373-y (PMC12949501; doi:10.1186/s42836-026-00373-y)
Supplement: Supplementary file 1 — Supplementary Material 1. [file 42836_2026_373_MOESM1_ESM.docx]

| **Category** | **Coding System** | **Codes Included** | **Description / Use in Study** |
| --- | --- | --- | --- |
| **Primary Total Knee Arthroplasty (TKA)** | ICD-10-PCS | 0SRC069, 0SRC06A, 0SRC06Z, 0SRC07Z, 0SRC0J9, 0SRC0JA, 0SRC0JZ, 0SRC0KZ, 0SRC0L9, 0SRC0LA, 0SRC0LZ, 0SRD069, 0SRD06A, 0SRD06Z, 0SRD07Z, 0SRD0J9, 0SRD0JA, 0SRD0JZ, 0SRD0KZ, 0SRD0L9, 0SRD0LA, 0SRD0LZ | Codes used to identify primary total knee arthroplasty procedures (cemented and cementless; right and left knee; open approach) |
| **Robotic-Assisted Surgery** | ICD-10-PCS | 8E0Y0CZ, 8E0YXCZ | Codes used to identify robotic-assisted total knee arthroplasty |
| **COVID-19 Infection (Exclusion)** | ICD-10-CM | U07.1 | Used to exclude admissions associated with COVID-19 infection |

**Supplementary Table S1. ICD-10 Codes Used for Cohort Definition and Exclusions**

**Supplementary Table S1.** ICD-10-PCS and ICD-10-CM codes used to define the study cohort, identify robotic-assisted procedures, and apply exclusion criteria in the Nationwide Readmissions Database.
